# Emotional Impact of Cardiopulmonary Resuscitation Training on High School Students

**DOI:** 10.3389/fpubh.2017.00362

**Published:** 2018-01-29

**Authors:** Abdullah Alismail, Evelyn Massey, Cassaundra Song, Noha Daher, Michael H. Terry, David López, Laren Tan, Takkin Lo

**Affiliations:** ^1^Cardiopulmonary Sciences, Loma Linda University, Loma Linda, CA, United States; ^2^Chaminade University of Honolulu, Honolulu, HI, United States; ^3^Allied Health Studies, Loma Linda University, Loma Linda, CA, United States; ^4^Respiratory Care, Loma Linda University Medical Center (LLUMC), Loma Linda, CA, United States; ^5^Loma Linda University Medical Center (LLUMC), Loma Linda, CA, United States; ^6^Castle Medical Center, Kailua, HI, United States

**Keywords:** cardiopulmonary resuscitation, heart arrest, sudden death, CPR, emotions, high school, students

## Abstract

**Background:**

The American Heart Association (AHA) has implemented several programs to educate the public about cardiopulmonary resuscitation (CPR). A common issue in bystander CPR is the fear of hurting the victim. As a result, the victim may not receive CPR in time. The purpose of this study was to measure the emotional impact of CPR training on high school students using two approved AHA courses.

**Methods:**

A total of 60 students participated in this study. These students had a mean age of 15.4 ± 1.2 years old and were selected from a high school in Southern California. Subjects were divided into two groups, Basic Life Support (BLS) (*n*_1_ = 31) and Hands-Only™ CPR (*n*_2_ = 29). Emotional impacts were assessed by having each subject answer a questionnaire based on given scenarios before and after their training session.

**Results:**

There was a significant difference in both groups when comparing positive-emotion scores before and after the training (BLS: 30.3 ± 6.0 vs. 34.5 ± 6.7, *p* < 0.001; Hands-Only 27.9 ± 5.0 vs. 32.1 ± 6.5, *p* < 0.001). In addition, both groups showed significant reductions in negative-emotion scores (BLS: 29.2 ± 6.7 vs. 23.7 ± 6.5, *p* < 0.001 and Hands-Only: 26.8 ± 6.1vs. 24.8 ± 7.7, *p* = 0.05).

**Conclusion:**

Our results indicate that the AHA programs have positive effects on students’ emotional response. We recommend that future studies include an in-depth study design that probes the complexity of students’ emotions after completing an AHA session.

## Introduction

According to a report published by the Center for Disease Control and Prevention, between 2005 and 2010, approximately 90% of individuals who experienced an out-of-hospital cardiac arrest (OHCA) died (1). According to this report, approximately 300,000 individuals suffered significant levels of morbidity and elevated mortality rates (1). The American Heart Association (AHA) states that nearly 70% of the American population may not know how to appropriately administer cardiopulmonary resuscitation (CPR) according to current AHA guidelines (2), that almost 70% of cardiac arrests occur at home (3), and that there were more than 350,000 OHCA incidents in the United States, with a survival rate of 9.5% (3, 4). Given that the majority of cardiac arrests occur outside of a hospital setting, more effective approaches to educating the public regarding CPR must be explored (5).

Most recently, the AHA has envisioned educating the public about CPR by implementing programs at schools, institutions, and organizations. One of these programs is “CPR in School.” In this program, the goal is to provide schools with a portable training kit that can be used to teach approximately 20 students in a given session. Current studies have shown the benefits of teaching high school-aged students and the general public Hands Only™ CPR and Basic Life Support (BLS) CPR (6–9). Hands Only™ CPR is a brief and simplified version of BLS. It consists of a technique in which only chest compression is administered, as compared to the typical BLS taught in hospital training, in which both ventilation and automatic external defibrillation (AED) are used.

Over time, teaching high school-aged students Hands Only™ CPR can increase the proportion of CPR-trained adults, as well as enhancing OHCA awareness (8, 10). In addition, high school-aged students are more likely to be physically fit and thus able to perform CPR correctly (10, 11). Providing CPR training to a broader swath of the population increases the chances of survival for OHCA victims. Cave et al. addressed the importance of implementing CPR training as a high school graduation requirement because this will improve OHCA response rates (11).

Meissner et al. (8) performed a longitudinal study in which they assessed students’ retention and confidence pre- and posttraining, as well as 4 months later. In their study, they report good knowledge retention among the tested group. In another prospective study, a junior physician provided a 45-min training session (6). This session was deemed more helpful and more convenient than a 4-h session, which had been used in prior studies. Simplifying BLS training to Hands Only™ CPR has been shown to increase public understanding of OHCA, as well as survival rates for OHCA victims, as compared to no training (12). Various studies have also shown that training high school students in BLS provides positive results regarding BLS, CPR, and AED knowledge post-training (7, 8).

Some reports suggest that many individuals fear the potential legal ramifications resulting from the provision of CPR. This fear of the legal implications of administering CPR and/or AED due to hurting the OHCA victim is a common challenge in bystander CPR (13–15). Studies show that one reason for this fear is a lack of knowledge regarding CPR (6, 16). One study reports that even people who are trained in BLS are reluctant to use AED (16). Reiner et al. (17) report on the spectrum of federal and state laws regarding the use of AED. In most of the United States, untrained individuals are permitted to use AED. However, in a few states, individual training is required (17). However, the researchers also report that rescuers in all states are protected by law after they administer CPR, including AED.

Savastano and Vanni (18) investigated “the most frequent fears” among lay rescuers after administering the AHA BLS course for family and friends and asked these individuals whether they would perform CPR in a real scenario. The fear of causing damage and the fear of not being able to perform CPR were the most significant fears reported by the participants. The researchers concluded that people are often afraid to implement CPR in real scenarios. This shows that the emotions, especially fear, affect a person’s willingness to perform CPR, especially if such a person lacks background knowledge regarding healthcare, such as a high school student (18–26). Aaberg et al. (6) also reported that fear was a common theme among tested high school students. Teaching one Hands-Only™ CPR session decreased fear levels significantly. Furthermore, Mpotos et al. (27) investigated the willingness of teachers who were BLS-certified to teach CPR to their students *via* a national survey. Surprisingly, more than half of their sample showed an unwillingness to teach CPR due to their lack of confidence and training (27).

The purpose of our study was to measure the emotional impact of CPR training on high school students quantitatively using the Positive and Negative Affect Schedule (PANAS) questionnaire (28). In this questionnaire, a total of 20 emotions (10 positive and 10 negative) are attached to a scenario (see Table A1 in Appendix 1). Subjects will respond to the presented scenario quantitatively. Our aim was to measure how attending an AHA session affected the students’ emotions.

## Materials and Methods

This study was approved by the Institutional Review Board at Loma Linda University, Loma Linda, CA, USA. The study subjects were students from a selected high school in Southern California, United States of America. The selected high school was enrolled in the CPR training sessions provided by an approved AHA training site. These training sessions were taught by certified AHA instructors. An informed consent and information documents were mailed to the high school students’ parents or legal guardians for approval. No identifiers were collected during the study. The study design was quasiexperimental. Subjects were included if they were high school students in grades 9–12. Subjects were excluded if they had had previous exposure to BLS and/or Hands-Only™ CPR. A total of 60 subjects with mean age of 15.4 ± 1.2 years were enrolled in the study. These subjects were then divided into two groups, the BLS group (*n*_1_ = 31) and the Hands Only™ CPR group (*n*_2_ = 29). Group selection was performed by the school administrators. The BLS group was composed of students in a health science track, and the Hands-Only group was composed of students on a non-health-science track. The research team did not affect the subjects’ assignment into the groups.

Both groups were given a folder that contained a demographics sheet and pre- and post-training questionnaires based on two written OHCA scenarios. Subjects were instructed to complete the pre- and post-training questionnaires and return them to an assigned researcher. The post-questionnaires were measured immediately after the subjects finished their sessions. The purpose of this PANAS questionnaire was to measure the subject’s mood and emotional levels quantitatively in light of the two written OHCA scenarios. The survey includes 20 emotions, 10 positive and 10 negative, and has been validated by Watson et al. (28).

The BLS group was instructed to attend a full BLS course. This course followed AHA guidelines, including a full didactic hands-on session and the teaching the use of an automated external defibrillator (course is 4 h long). The second group completed the Hands Only™ CPR course, which also followed AHA guidelines. In the Hands Only™ CPR group, subjects were only asked to perform the AHA process steps in response to a cardiac arrest which is faster and shorter than the BLS course. These steps were (1) call 9-1-1 and (2) push hard and fast (Chest compression) without any AED or fully didactic course component (29).

The two scenarios were as follows:

*Scenario 1*: You are playing basketball with your friends in the gym. Suddenly, one of the players collapses and is not breathing or responding to you. You check his pulse and do not find one. You tell a friend to call 911 and immediately begin chest compressions. In this situation, how strongly do you feel each of the following emotions?
*Scenario 2*: You are standing at a bus stop, and an adult collapses next to you. He is not breathing or responding to you. You do not feel a pulse; you yell for someone to call 911. There is an automatic external defibrillator at the bus stop. You immediately start chest compressions while someone sets up the automated external defibrillator to deliver a shock to the victim. In this situation, how strongly do you feel each of the following emotions?

### Data Analysis

A sample size of 66 participants (33 per group) was estimated using a medium effect size of 0.7, a power of 0.8, and an alpha of 0.05. However, we were able to enroll only 60 participants. The data were summarized using frequencies and relative frequencies for qualitative variables and means ± SDs for quantitative variables. To compare the distribution of the categorical variables between the two study groups, a chi-square test was used. Due to the inclusion of two scenarios that could arouse both negative and positive emotions, the average scores for these scenarios were computed. We also calculated the difference in the answers between pretraining and post-training, along with a 95% confidence interval (CI). The normality of the positive and negative emotion scores pre- and post-training, as well as the differences between them, were examined using histograms and box-and-whisker plots. For each group, changes (pre- versus post-) were assessed using paired *t*-tests. Because the distribution of the difference between pre- and post-training was approximately normal, an independent *t*-test was used to examine this difference by study group. We also conducted an analysis of covariance to compare pre- versus post-training scores by study group while controlling for the scores at baseline. The level of significance was set at a *p*-value ≤0.05.

## Results

Sixty high school students participated in this study and were allocated into either the BLS group (*n*_1_ = 31) or the Hands Only™ CPR group (*n*_2_ = 29). There were no significant differences between the two study groups in terms of gender, education level, or mean age (*p* > 0.05, Table 1). There were no significant differences in mean baseline positive and negative emotion scores between the BLS and Hands Only™ CPR training groups (30.3 ± 6.0 vs. 27.9 ± 5.0, *p* = 0.10; and 29.2 ± 6.7 vs. 26.8 ± 6.1, *p* = 0.14, respectively).

**Table 1:** Frequency (%) of general characteristics of subjects by study group (*N* = 60).

	BLS (*n*_1_ = 31)	Hands-Only (*n*_2_ = 29)
Age (years, mean ± SD)	15.3 ± 1.2	15.6 ± 1.2
**Gender**		
Male	3 (9.7)	6 (20.7)
Female	18 (58.1)	18 (62.1)
Missing^a^	10 (32.3)	5 (17.2)
**Grade level**		
Grade 9	6 (19.4)	6 (20.7)
Grade 10	9 (29.0)	3 (10.3)
Grade 11	8 (25.8)	8 (27.6)
Grade 12	6 (19.4)	7(24.1)
Missing^a^	2 (6.5)	5 (17.2)

*^a^Several subjects missed answering the question*.

*BLS, Basic Life Support*.

There was a significant increase in the mean positive emotion score for both the BLS and Hands Only™ CPR training groups (*p* < 0.001, Table 2). Negative emotions were significantly reduced in both the BLS (*p* < 0.001) and Hands Only™ CPR groups (*p* = 0.05, Table 2). The results of the independent t-test showed that the improvement in positive emotions was not significantly different between the groups [mean difference (95% CI); 0.1 (−2.3, 2.5), *p* = 0.92, η^2^ = 0.1]. However, the decreases in negative emotions were significantly different between the groups, with the BLS group undergoing a greater decrease [mean difference (95% CI); −3.6 (−6.3, −0.90), *p* = 0.01, η^2^ = 0.7].

**Table 2:** Means and SDs for positive and negative emotions by study group.

Emotions	BLS (*n*_1_ = 31)			Hands-Only (*n*_2_ = 29)		
	Pre	Post	*p*-Value	Pre	Post	*p*-Value
Positive emotions	30.3 ± 6.0	34.4 ± 6.7	<0.001	27.9 ± 5.0	32.1 ± 6.5	<0.001
Negative emotions	29.2 ± 6.7	23.7 ± 6.5	<0.001	26.8 ± 6.1	24.8 ± 7.7	0.05

*BLS, Basic Life Support*.

The analysis of covariance revealed that the positive emotion scores improved over time (*F*_1,57_ = 6.5, *p* = 0.01) and that there was no significant interaction between these scores and time (*F*_1,57_ = 1.6, *p* = 0.20). Regarding the negative emotion scores, however, there was a significant interaction between the scores and time (*F*_1,57_ = 5.3, *p* = 0.03), which was consistent with the finding from the independent *t*-test.

## Discussion

The objective of this study was to assess feelings about OHCA events among high school students by administering a survey before and after BLS and Hands-Only™ CPR courses.

According to Watson et al. (28), who developed and validated the PANAS scale, the expected momentary means are 29.7 for positive emotions and 14.8 for negative emotions. The results are interpreted differently for these two different types of emotions. For positive emotions, if the score for the 10 positive emotions is increased after the course session, this means that subjects have experienced increased positive emotions. For negative emotions, if the score is less negative after the training, this means that the subject is having a more positive emotional response, or a less negative emotional response, to the presented scenarios after the session (28).

Comparing the momentary means given by Watson et al. (28) and our results, it is clear that both groups underwent a significant increase in their mean scores for positive emotions. This indicates that these high school students will feel more confident in responding to a cardiac arrest scenario post-training. When comparing our negative emotion scores to Watson’s et al. (28), both of our groups had higher mean values than the proposed momentary score of 14.8. However, the BLS group showed a more significant decrease in their negative emotions as compared to the Hands-Only group. One potential explanation for this finding is that the Hands Only™ CPR group did not complete a full didactic course, in contrast to the BLS group, in which automated external defibrillator use was taught and discussed in detail. Teaching automated external defibrillator use to all students will likely improve their positive and negative emotional responses, as seen in this study. More confident rescuers will be better able assess the victim’s heart rhythm and take action until healthcare providers arrive to the scene. Our data supports and reinforces the AHA’s current mission of expanding access to and knowledge of automated external defibrillator use in public areas and schools due to its significant effect on rescuers’ confidence and positive emotions.

Watson et al. (30) compared the use of a medical emergency plan and the prevalence of automated external defibrillator placement in Tennessee high schools before and after legislation. In their study, they found an increased number of medical emergency plans and automated external defibrillator placements in schools from 2006 to 2011. However, they also noticed a decrease in CPR training (30). Given the results of our study, we believe that high school students’ emotional responses will change based on the type of CPR training they receive. Furthermore, Drezner et al. (31) conducted a 2-year prospective observational study of schools that were registered in an AED-based program. Based on their data, they found a strong increase in the rate of response to sudden cardiac arrests. The survival rate of sudden cardiac arrest victims from these schools was very high (80% if the school had and used an AED versus 50% if it was brought by EMS; *p* = 0.03) (31). Furthermore, in a study performed in a Danish high school, researchers evaluated the effectiveness of a junior doctor teaching high school students how to be first responders to OHCA (6). Their results showed a significant increase in the students’ knowledge after the training session. In our study, we examined the emotional responses of high school students’ pre- and post-training. Our results show that there is a strong emotional response after the AHA training session.

This study used a valid and reliable quantitative measurement tool for positive and negative emotional response before and after AHA training sessions (28). To our knowledge, we are the first to measure this type of emotions (positive and negative), before and after AHA training courses using the PANAS survey. Our findings further support the AHA’s goal of providing and promoting programs that educate the community and thus decrease the incidence of OHCA.

In support to the findings of this study, it was reported to our research team that one of the subjects who participated in the study witnessed an OHCA event involving a family member. The subject immediately called 911 and applied the CPR training detailed in this study until the paramedics arrived. As a consequence of this action, the subject saved the family member’s life.

### Limitations

The sample used in this study was a judgment sample, and the participants were assigned to the groups based on an administrator’s decision, which may indicate selection bias. Hence, we recommend that further studies randomly assign participants to various groups. We believe that an in-depth study that includes interviews with focus groups is a better way to understand emotions. We thus recommend that future researchers conduct an in-depth qualitative study probing the complexities of the emotions that appear when performing OHCA. This approach will provide an in-depth thematic structure and a theoretical framework that can better explain the emotional responses involved.

## Conclusion

In this study, there was a significant improvement in the subjects’ positive and negative emotional responses after an AHA session. Due to the education that was provided to these high school students, one of them saved a life. This supports the benefits of having programs such as BLS and Hands-Only™ CPR in schools. We believe that these programs are beneficial to young students and will allow them to contribute to their community in the future. Further research that probes the complexities of the emotions involved in CPR is needed.

## Ethics Statement

This study was approved by the Institutional Review Board (IRB) at Loma Linda University, Loma Linda, CA, USA. The IRB that approved the study waived the need for written and informed consent to be obtained from the parents of the participants and requested only parental notification. Parental notification was done by mailing them a letter from the study PI. The parents and kids had the right to refuse participation.

## Author Contributions

AA: study design, data collection, interpretation and analysis, and manuscript writing; EM: CO-PI, study design, data collection, recruitment and overseeing the project, and assuring both CPR administered courses followed AHA guidelines; CS: study design, data collection, and manuscript writing; ND: statistician and manuscript writing; MT: study design and manuscript writing, and editing; DL and LT: manuscript writing, editing, and interpretation; TL: principle investigator, study design, interpretation, and overseeing the project.

## Conflict of Interest Statement

The authors declare that the research was conducted in the absence of any commercial or financial relationships that could be construed as a potential conflict of interest. The reviewer MK and handling editor declared their shared affiliation.

## Appendix 1

**Table A1:** Positive and Negative Affect Schedule questionnaire used to measure emotions and mood before and after the cardiopulmonary resuscitation training session.

Emotion	Very slightly or not at all	A little	Moderately	Quite a bit	Extremely
Interested	1	2	3	4	5
Distressed	1	2	3	4	5
Excited	1	2	3	4	5
Upset	1	2	3	4	5
Strong	1	2	3	4	5
Guilty	1	2	3	4	5
Scared	1	2	3	4	5
Hostile	1	2	3	4	5
Enthusiastic	1	2	3	4	5
Proud	1	2	3	4	5
Irritable	1	2	3	4	5
Alert	1	2	3	4	5
Ashamed	1	2	3	4	5
Inspired	1	2	3	4	5
Nervous	1	2	3	4	5
Determined	1	2	3	4	5
Attentive	1	2	3	4	5
Jittery	1	2	3	4	5
Active	1	2	3	4	5
Afraid	1	2	3	4	5

*The questionnaire included 10 positive and 10 negative emotions (a total of 20). Subjects rated how greatly they felt each emotion based on a given scenario. Highlighted emotions are positive, and non-highlighted emotions are negative*.
